# Supplementary material for: IL-33 promotes transcriptional and metabolic adaptations of tissue-resident Th2 cells
Source: J Immunol. 2026 Apr 16;215(4):vkag028. doi: 10.1093/jimmun/vkag028 (PMC13082907; doi:10.1093/jimmun/vkag028)
Supplement: vkag028_Supplementary_Data [file vkag028_supplementary_data.zip › Th2.revision.SUP.figures.with.legends.v5.pdf]

Sup. Fig.1

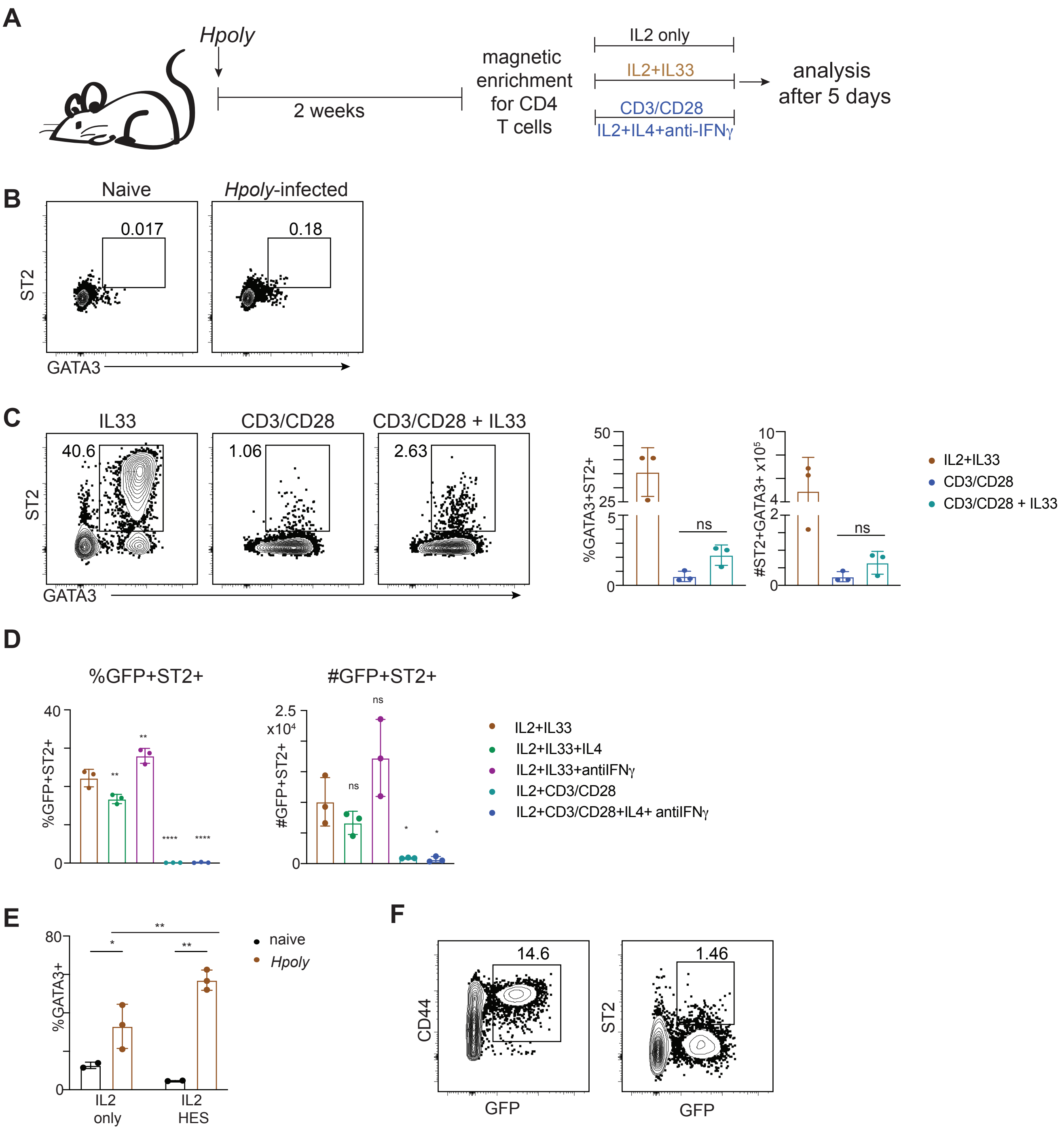
A
Hpoly
2 weeks
magnetic enrichment for CD4 T cells
IL2 only
IL2+IL33
CD3/CD28
IL2+IL4+anti-IFNγ
analysis after 5 days
B
Naive
Hpoly-infected
0.017
0.18
ST2
GATA3
C
IL33
CD3/CD28
CD3/CD28 + IL33
40.6
1.06
2.63
ST2
GATA3
%GATA3+ST2+
#ST2+GATA3+ x10^5
ns
ns
IL2+IL33
CD3/CD28
CD3/CD28 + IL33
D
%GFP+ST2+
#GFP+ST2+
x10^4
ns
IL2+IL33
IL2+IL33+IL4
IL2+IL33+antiIFNγ
IL2+CD3/CD28
IL2+CD3/CD28+IL4+ antiIFNγ
E
%GATA3+
naive
Hpoly
IL2 only
IL2 HES
F
14.6
1.46
CD44
ST2
GFP
GFP

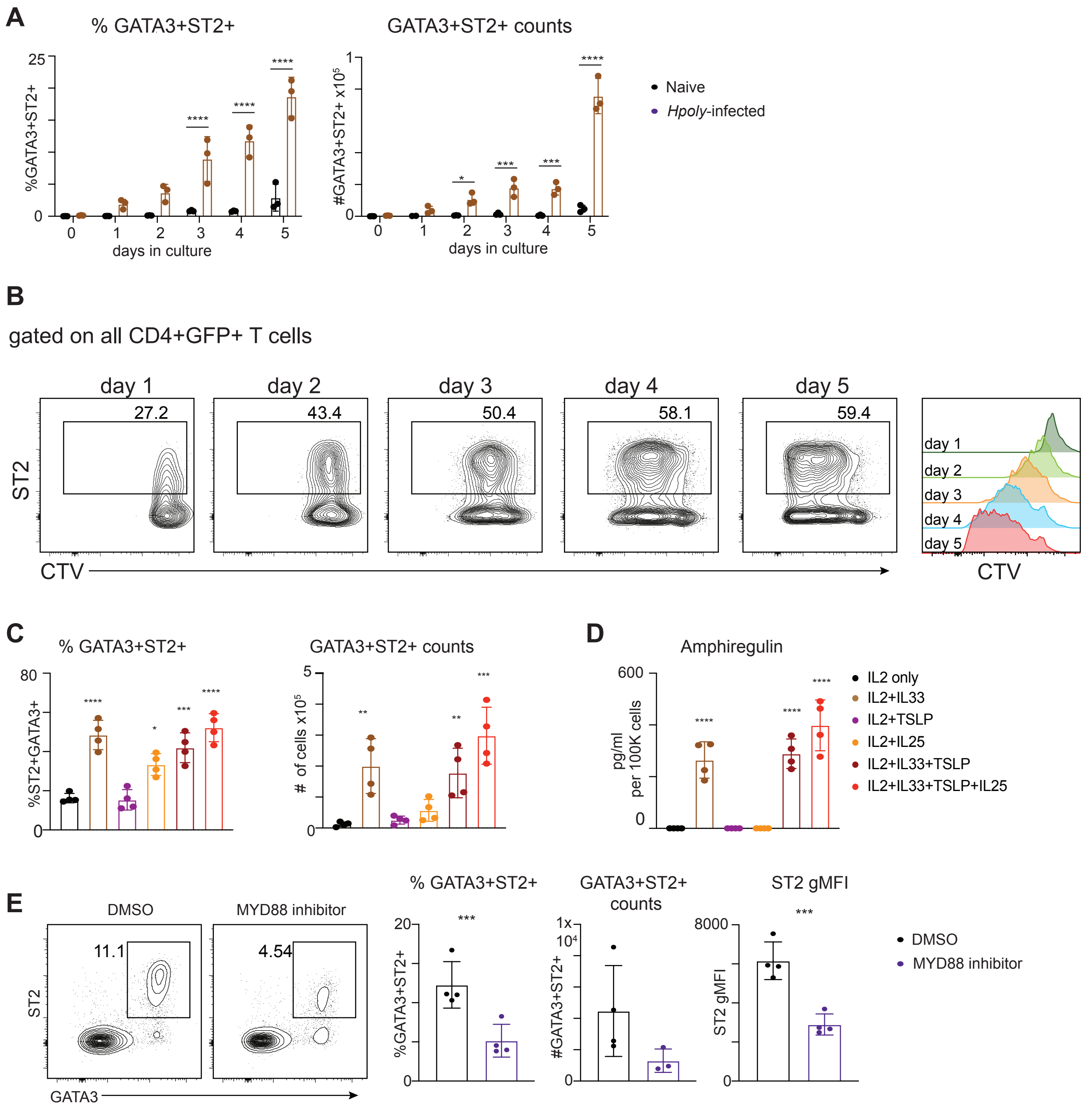
A
% GATA3+ST2+
%GATA3+ST2+
days in culture
GATA3+ST2+ counts
#GATA3+ST2+ x10^5
days in culture
Naive
Hpoly-infected
B
gated on all CD4+GFP+ T cells
day 1
day 2
day 3
day 4
day 5
27.2
43.4
50.4
58.1
59.4
ST2
CTV
CTV
C
% GATA3+ST2+
%ST2+GATA3+
GATA3+ST2+ counts
# of cells x10^5
D
Amphiregulin
pg/ml per 100K cells
IL2 only
IL2+IL33
IL2+TSLP
IL2+IL25
IL2+IL33+TSLP
IL2+IL33+TSLP+IL25
E
DMSO
MYD88 inhibitor
11.1
4.54
ST2
GATA3
% GATA3+ST2+
%GATA3+ST2+
GATA3+ST2+ counts
#GATA3+ST2+
ST2 gMFI
ST2 gMFI
DMSO
MYD88 inhibitor

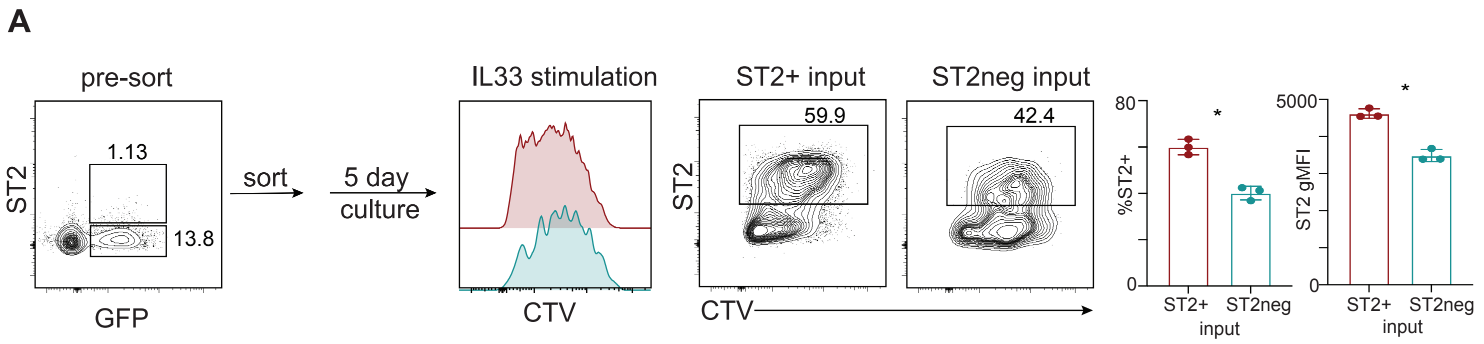
A
pre-sort
ST2
1.13
13.8
GFP
sort
5 day
culture
IL33 stimulation
CTV
ST2+ input
ST2neg input
59.9
42.4
ST2
CTV
80
0
%ST2+
*
ST2+
ST2neg
input
5000
0
ST2 gMFI
*
ST2+
ST2neg
input

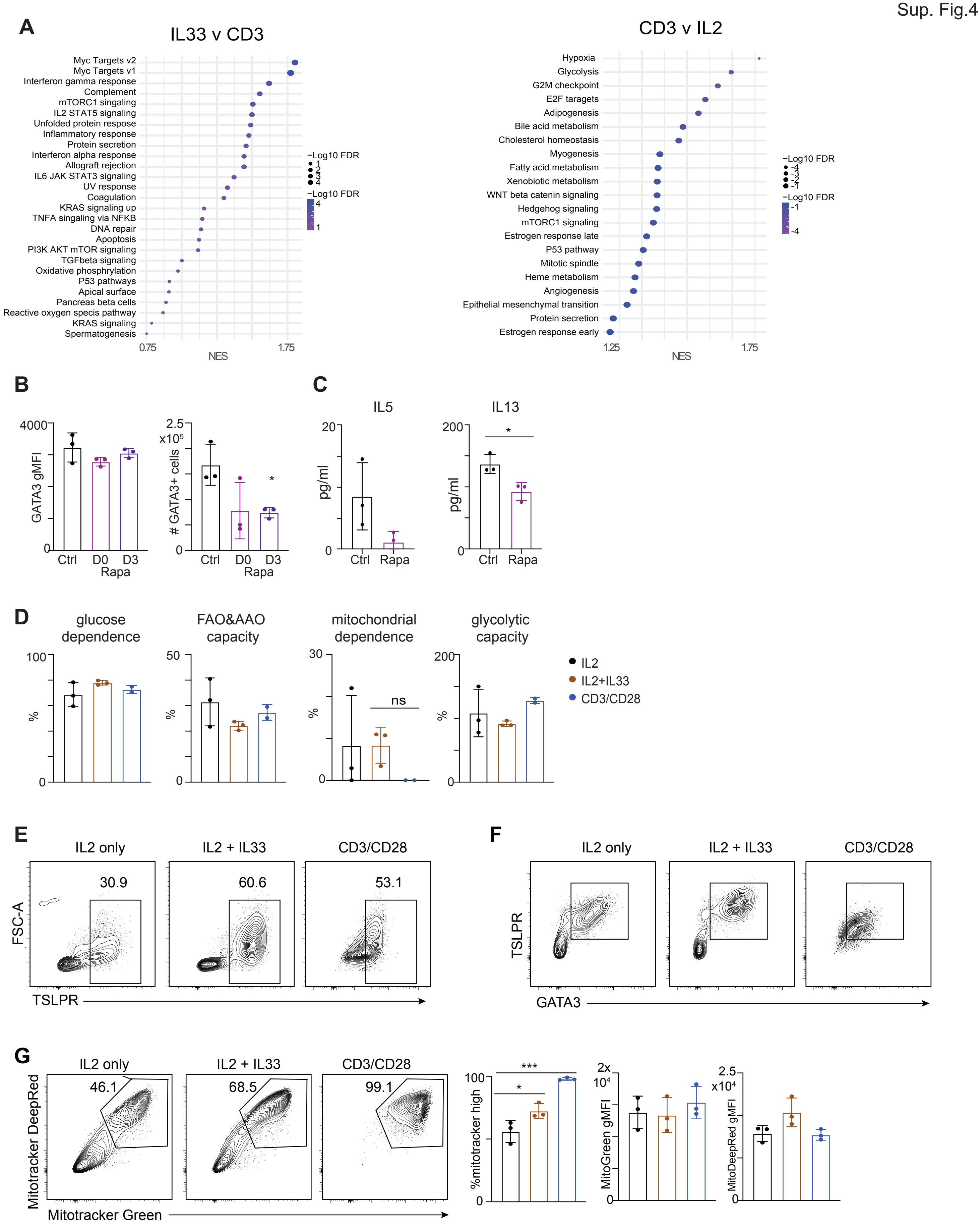
A
IL33 v CD3
Myc Targets v2
Myc Targets v1
Interferon gamma response
Complement
mTORC1 singaling
IL2 STAT5 signaling
Unfolded protein respose
Inflammatory response
Protein secretion
Interferon alpha response
Allograft rejection
IL6 JAK STAT3 signaling
UV response
Coagulation
KRAS signaling up
TNFA singaling via NFKB
DNA repair
Apoptosis
PI3K AKT mTOR signaling
TGFbeta signaling
Oxidative phosphrylation
P53 pathways
Apical surface
Pancreas beta cells
Reactive oxygen specis pathway
KRAS signaling
Spermatogenesis
NES
CD3 v IL2
Hypoxia
Glycolysis
G2M checkpoint
E2F taragets
Adipogenesis
Bile acid metabolism
Cholesterol homeostasis
Myogenesis
Fatty acid metabolism
Xenobiotic metabolism
WNT beta catenin signaling
Hedgehog signaling
mTORC1 signaling
Estrogen response late
P53 pathway
Mitotic spindle
Heme metabolism
Angiogenesis
Epithelial mesenchymal transition
Protein secretion
Estrogen response early
−Log10 FDR
B
GATA3 gMFI
GATA3+ cells
Ctrl D0 D3
Rapa
C
IL5
IL13
pg/ml
Ctrl Rapa
D
glucose dependence
FAO&AAO capacity
mitochondrial dependence
glycolytic capacity
ns
IL2
IL2+IL33
CD3/CD28
E
IL2 only
IL2 + IL33
CD3/CD28
30.9
60.6
53.1
FSC-A
TSLPR
F
TSLPR
GATA3
G
46.1
68.5
99.1
Mitotracker DeepRed
Mitotracker Green
%mitotracker high
MitoGreen gMFI
MitoDeepRed gMFI

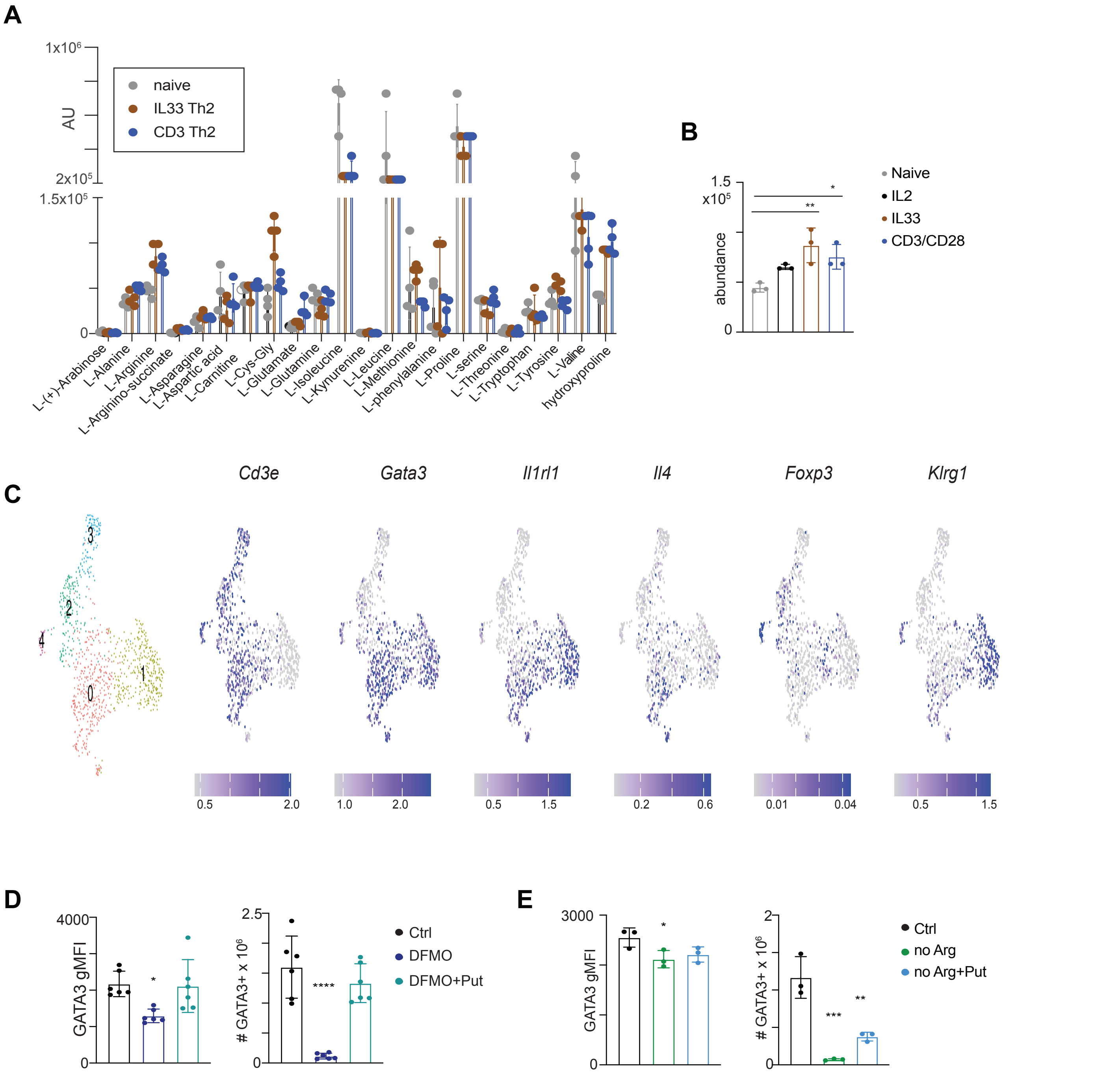
A
1x10⁶
AU
2x10⁵
1.5x10⁵
0
naive
IL33 Th2
CD3 Th2
L-(+)-Arabinose
L-Alanine
L-Arginine
L-Arginino-succinate
L-Asparagine
L-Aspartic acid
L-Carnitine
L-Cys-Gly
L-Glutamate
L-Glutamine
L-Isoleucine
L-Kynurenine
L-Leucine
L-Methionine
L-phenylalanine
L-Proline
L-serine
L-Threonine
L-Tryptophan
L-Tyrosine
L-Valine
hydroxyproline
B
1.5
x10⁵
abundance
0
Naive
IL2
IL33
CD3/CD28
C
Cd3e
Gata3
Il1rl1
Il4
Foxp3
Klrg1
0.5
2.0
1.0
2.0
0.5
1.5
0.2
0.6
0.01
0.04
0.5
1.5
D
4000
GATA3 gMFI
0
2.5
GATA3+ x 10⁶
0
Ctrl
DFMO
DFMO+Put
E
3000
GATA3 gMFI
0
2
GATA3+ x 10⁶
0
Ctrl
no Arg
no Arg+Put

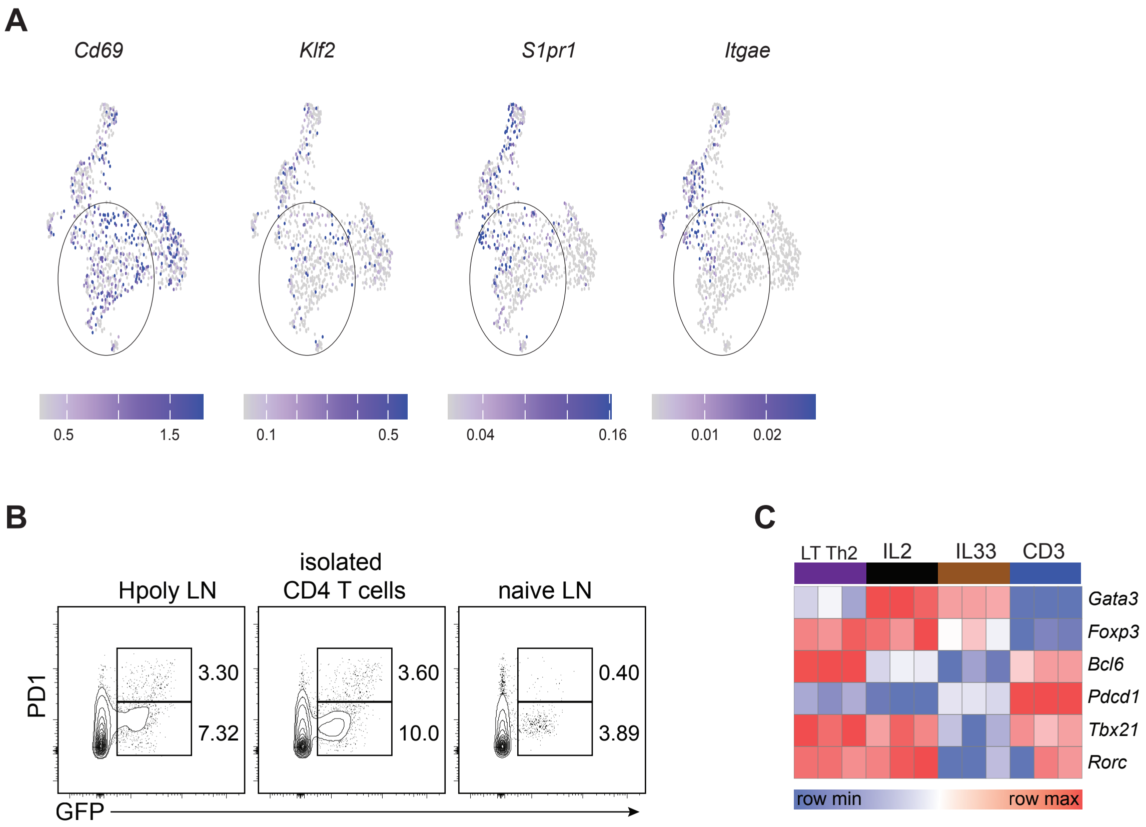
A
Cd69
Klf2
S1pr1
Itgae
0.5
1.5
0.1
0.5
0.04
0.16
0.01
0.02
B
Hpoly LN
isolated CD4 T cells
naive LN
PD1
GFP
3.30
7.32
3.60
10.0
0.40
3.89
C
LT Th2
IL2
IL33
CD3
Gata3
Foxp3
Bcl6
Pdcd1
Tbx21
Rorc
row min
row max

Sup. Fig. 1 Characterization of culture conditions. (A) A schematic of experimental design. CD4 T cells were isolated from *Hpoly*-infected mice and cultured with IL2 alone, IL2 and IL-33, or anti-CD3/CD28 under Th2 polarizing conditions for five days. (B) Representative flow plot of CD4+ T cells immediately following isolation from secondary lymphoid organs of naïve or *Hpoly*-infected mice. (C) CD4 T cells from secondary lymphoid organs of *Hpoly*-infected mice were cultured under the indicated conditions. Frequencies and numbers of GATA3+ST2+ cells were quantified. (D) CD4 T cells from secondary lymphoid organs of *Hpoly*-infected 4get mice were cultured under the indicated conditions. Frequencies and numbers of GFP+(IL4+)ST2+ cells were quantified. (E) Splenocytes from naïve or Hpoly-infected mice cultured with IL2 alone or together with HES. Frequencies of GATA3+ cells were quantified. (F) Representative flow plot of CD4+ T cells right after isolation from secondary lymphoid organs of *Hpoly*-infected 4get mice. The data (B-F) are representative of at least two independent experiments. Symbols in the quantified data represent independent biological replicates. Data were analyzed by one-way ANOVA with Tukey's post hoc test (C,D) or two-way ANOVA with Sidak post hoc test (E). * $p \leq 0.05$, ** $p \leq 0.01$, *** $p \leq 0.001$.

Sup. Fig. 2 IL-33 is the primary driver of Th2$_{TRL}$ cells. (A) CD4+ T cells from naïve or *Hpoly*-infected mice were cultured with IL2 and IL-33. Frequencies and numbers of GATA3+ST2+ cells were quantified over five days. (B) CD4+ T cells from *Hpoly*-infected 4get mice were stained with CTV and cultured with IL2 and IL-33. Representative flow plots and histograms of ST2 expression and CTV dilatation over the course of five days. (C) CD4+ T cells from *Hpoly*-infected mice were cultured under the indicated conditions. Frequencies and numbers of GATA3+ST2+ cells were quantified after five days. (D) Abundance of amphiregulin in the culture supernatants from (C). (E) CD4+ T cells from *Hpoly*-infected mice were cultured with IL2 and IL-33 in the presence of 1μm MYD88 inhibitor or vehicle control for five days. Frequencies and number of GATA3+ST2+ cells as well as ST2 gMFI were quantified. The data (A-E) are representative of at least two independent experiments. Symbols in the quantified data represent independent biological replicates. Data were analyzed by one-way ANOVA with Tukey's post hoc test (C), two-way ANOVA with Sidak post hoc test (A), or paired T-test (E). * $p \leq 0.05$, ** $p \leq 0.01$, *** $p \leq 0.001$.

Sup. Fig. 3 CD4 T cells from *Hpoly*-infected mice are primed to expand *in vitro*. (A) CTV-labelled IL4+ (GFP)+ ST2+ and IL4+(GFP+)ST2^{NEG} early Th2 cells were FACS-isolated from 4get mice infected with *Hpoly* and cultured with IL2 and IL-33 for five days. Frequencies of ST2+ cells were quantified. The data are representative of at least two independent experiments. Symbols in the quantified data represent independent biological replicates. Data were analyzed by paired T-test (D). * $p \leq 0.05$, ** $p \leq 0.01$, *** $p \leq 0.001$.

Sup. Fig. 4. Metabolic adaptations of Th2 cells. (A) Top Hallmark pathways from GSEA for IL-33 v CD3 and CD3 v IL2 comparisons. (B) CD4+ T cells were isolated from *Hpoly*-infected mice and cultured with anti-CD3/CD28 under Th2 polarizing conditions in the presence of rapamycin. The mean fluorescence intensity of GATA3 and cell numbers were quantified. (C) Levels of indicated cytokines after overnight culture of anti-CD3/CD28-stimulated cells in fresh media containing rapamycin. (D) Quantification of results from SCENITH assay in GATA3+ Th2 cells. Representative flow plot showing the expression of TSLPR (E) and TSLPR and GATA3 (F) expression after five days of *in vitro* culture under the conditions shown, of CD4+ T cells isolated from *Hpoly*-infected mice. (G) Representative flow plot and quantification of mitotracker green and mitotracker deep red

staining after five days of culture under the indicated conditions. The data (B-F) are representative of at least two independent experiments. Symbols in the quantified data represent independent biological replicates. Data were analyzed by one-way ANOVA with Tukey's post hoc test (B, D, G), or paired T-test (C). * $p \leq 0.05$, ** $p \leq 0.01$, *** $p \leq 0.001$.

Sup. Fig. 5. Amino acid metabolism in Th2 cells. (A) Intracellular abundance of amino acids was measured by LC/MS in naïve, IL33-stimulated, and anti-CD3/CD28-stimulated CD44+CD4+ T cells after five days of culture. (B) Intracellular abundance of arginine in naïve cells, IL2-stimulated, IL33-stimulated, and anti-CD3/CD28-stimulated CD44+ CD4+ T cells after five days of culture. (C) Expression of Th2 cell-related genes in scRNAseq dataset of T cells isolated from mAT of *Hpoly*-infected mice, identifying a cluster of GATA3+ST2+ Th2 cells (circled). CD4+ T cells from Hpoly-infected mice were stimulated with anti-CD3/CD28 and treated with DFMO (D) or cultured in arginine-depleted media (E). Mean fluorescence intensity of GATA3 and cell numbers were quantified. The data (A, D, E) are representative of at least two independent experiments. Symbols in the quantified data represent independent biological replicates. Data were analyzed by one-way ANOVA with Tukey's post hoc test (B,D,E). * $p \leq 0.05$, ** $p \leq 0.01$, *** $p \leq 0.001$.

Sup. Fig. 6. IL33 promotes tissue residency (A) Expression of genes associated with tissue-residency in scRNAseq dataset of isolated from mAT of *Hpoly*-infected mice, identifying cluster of GATA3+ST2+ Th2 cells (circled). (B) Representative flow plot of PD1 and GFP expression in naïve and *Hpoly*-infected 4get mice. (C) Heatmap depicting the expression of master transcription factors of major CD4+ T cell subsets, and of *Pdcd1*.
